# Evaluation of the Potential Entomopathogenic Fungi *Purpureocillium lilacinum* and *Fusarium verticillioides* for Biological Control of *Forcipomyia taiwana* (Shiraki)

**DOI:** 10.3390/jof8080861

**Published:** 2022-08-16

**Authors:** Nian-Tong Ni, Sing-Shan Wu, Kuei-Min Liao, Wu-Chun Tu, Chuen-Fu Lin, Yu-Shin Nai

**Affiliations:** 1 Department of Entomology, National Chung Hsing University, Taichung 402, Taiwan; 2 National Mosquito-Borne Diseases Control Research Center, National Health Research Institutes, Tainan 704, Taiwan; 3 Department of Veterinary Medicine, College of Veterinary Medicine, National Pingtung University of Science & Technology, Pingtung 921, Taiwan

**Keywords:** biting midge, *Forcipomyia taiwana*, entomopathogenic fungi, *Purpureocillium*, *Fusarium*

## Abstract

*Forcipomyia taiwana* (Diptera: Ceratopogonidae) is a nuisance blood-sucking pest to humans in Taiwan. An *F. taiwana* bite causes itching and redness and usually causes serious harassment to human outdoor activity. In terms of *F. taiwana* control, chemical pesticides are ineffective. Therefore, other efforts are needed. Fungal mycosis in the larvae, pupae, and emerging *F. taiwana* adults was found during the rearing of *F. taiwana*. In this study, six fungal isolates were isolated from infected cadavers and subjected to molecular identification. In addition, their biocontrol potential was evaluated against different life stages of *F. taiwana.* Based on the pathogenicity screening, two fungal isolates, NCHU-NPUST-175 and -178, which caused higher mortality on the fourth instar larvae of *F. taiwana*, were selected for virulence tests against different life stages of *F. taiwana* larvae. The results of the phylogenetic analysis indicated that the NCHU-NPUST-175 and -178 belonged to *Purpureocillium lilacinum* and *Fusarium verticillioides*, respectively. Bioassay against different life stages of *F. taiwana* with different spore concentrations (5 × 10^5^ to 5 × 10^7^ conidia/mL) revealed a dose-dependent effect on larvae for both fungal isolates, while only 38% and 50% mortality was found in highest concentration (5 × 10^7^ conidia/mL) at fourth instar larvae by Pl-NCHU-NPUST-175 and Fv-NCHU-NPUST-178, respectively. Moreover, reductions in egg-hatching rate and adult emergence rate were found, when the last stage of *F. taiwana* was inoculated with both fungal isolates, indicating the ovicidal potential and the impact of entomopathogenic fungi on the development of *F. taiwana*. In conclusion, Pl-NCHU-NPUST-175 and Fv-NCHU-NPUST-178 showed larvicidal activity, ovicidal activity, and impact on adult emergence on *F. taiwana*.

## 1. Introduction

*Forcipomyia taiwana* (Diptera: Ceratopogonidae) was first recorded and named in central Taiwan by Shiraki in 1913. *F. taiwana* is a harassing pest that sucks the blood during the daytime [1]. *F. taiwana* females bite humans and digest blood for the formation and development of their eggs [2]. Although there is no case of *F. taiwana* spreading disease that makes humans sick, *F. taiwana* biting can cause intense itching and redness in sensitive individuals [3] and the level of harassment has caused harm to people’s quality of life and tourism and leisure activities. Therefore, it is important to develop an effective control strategy to control *F. taiwana*.

The options for *F. taiwana* control are limited and the main strategy is environmental management. Although chemical pesticides are still the main method for controlling *F. taiwana* adults, the use of chemical pesticides is not an effective way to kill them and will harm the environment. In addition, the chemical pesticides can only control the adults, not all the life stages of *F. taiwana*; therefore, the development of other effective control methods is necessary for *F. taiwana* control [4].

In terms of the dipteran pest control, entomopathogenic fungi (EPFs) were discovered in wild mosquito populations. It has been found that numerous entomopathogenic fungi have insecticidal effects on different species of midges. For example, *Beauveria bassiana* and *Metarhizium anisopliae* were used to control *Culicoides nubeculosus* [5]. In addition, the fungi belonging to the genera *Purpureocillium* and *Fusarium* have been found in mosquito larvae and the insecticidal effect was also proved [6]. On the other hand, *Bacillus thuringiensis* var. *israelensis* has also been found to kill dipteran pests effectively [7]. As aforementioned, microbial control has the potential to control *F. taiwana*. However, there are still many problems for the application of EPFs in the field, such as high-temperature environment, dry condition, and ultraviolet light, etc. [8,9]. Therefore, it is necessary to select the fungal strains that have adapted to the environment and accommodate for combating survival adversity.

The genus *Purpureocillium* is a common entomopathogenic fungus and some species, such as *Purpureocillium lilacinum,* are commonly used to manage root knot nematodes (*Meloidogyne* spp.) and insect pests, such as *Aphis gossypii*, indicating the potential for pest control [10]. Though *Fusarium* fungi are considered as phytopathogenic fungi, there are more than 13 *Fusarium* fungi showing insect-killing activity against the Coleoptera, Diptera, Hemiptera, Hymenoptera, Lepidoptera, and diptera insects [11,12,13]. In this study, during the rearing of *F. taiwana* larvae, symptoms of mycosis were found in the larvae and pupae life stages. The fungi were isolated and identified based on the combination of morphological characteristics and molecular data. Then, the isolated fungi were subjected to tests on their virulence against different life stages of *F. taiwana,* both in the laboratory and in the field. The possible application strategy was also discussed.

## 2. Materials and Methods

### 2.1. Forcipomyia Taiwana Collection and Rearing

Adult *F. taiwana* females were collected by aspirator from the field using two layers of stockings on the human calf to attract them in Taichung, Taiwan. The captured adult females of *F. taiwana* were kept in net cages (10 cm × 10 cm), fed with 10% sucrose solution, and transferred to the laboratory in 12 h. The artificial blood-feeding apparatus devised by Luo (2018) was used to feed the females of *F. taiwana*. Three days after blood feeding, moist filter paper was provided for oviposition of *F. taiwana* in net cages for two days and then the filter paper was placed on a 9 cm 1.0% agar plate at room temperature for experiments.

### 2.2. Fungal Isolation and Selection

During the rearing of *F. taiwana* in the laboratory, fungal mycosis was found in the larvae, pupae, and emerging *F. taiwana* adults. The affected life stages (last stage of larvae and emerging *F. taiwana* adults) were collected and soaked in 75% ethanol for 3 min, placed separately in Eppendorf tubes with 500 μL phosphate buffer saline (pH 7.4) and homogenized. Then 100 μL of each homogenized suspension was taken and spread onto quarter-strength Sabouraud Dextrose Agar (1/4 SDA) in 90 mm Petri plates. The plates were incubated at 25 °C for 10 days to allow for fungal growth. Totally, six fungal isolates (two fungal isolates from last stage of larvae and four from emerging adults) were purified using single-spore method and stored in SDA slants at 4 °C for further studies. Pathogenicity screening was based on the *Tenebrio molitor*-mediated system followed by the method from Tettor-Barsch et al. and others [11,12,13,14,15]. The mortality of treated *T. molitor* larvae was observed and recorded daily for 10 days. The pathogenicity screening was repeated six times for each fungal isolate.

### 2.3. Molecular Identification

Fungal isolates were cultured on ¼ SDA medium in 60 mm Petri plates for 10 days and fungal genomic DNA was extracted from the hyphae using a Yeast Genomic DNA Kit (Geneaid, New Taipei, Taiwan) following the manufacturer’s instruction. Two primer sets were used for PCR amplification and sequencing, including the internal transcribed spacers (ITS1-5.8S-ITS2) and part of beta-tubulin gene [16,17] (Supplementary Table S1). The PCR mixture contained 10 μL of 2X SuperRed Master Mix (Tools, Taiwan), 1 μL of each primer, 1 μL of fungal genomic DNA, and 7 μL of ddH_2_O in a 20 μL reaction volume. PCR was performed using a thermal cycler (ABI Perkin Elmer 9700, USA) and the ITS region and beta-tubulin gene were amplified with the following reaction parameters: an initial cycle of denaturation at 95 °C for 10 min, followed by 35 cycles of denaturation at 95 °C for 15 s, annealing at 52 °C for 30 s and extension at 72 °C for 90 s, and a final extension step of 72 °C for 10 min. The PCR products were analyzed by electrophoresis using a 1% agarose gel in TAE buffer and purified using a GenepHlow™ Gel/PCR kit (Geneaid, New Taipei, Taiwan). The purified PCR products were sent to a sequencing service company (Genomics Co., New Taipei, Taiwan) for sequencing. The sequences were uploaded to NCBI GenBank database for identification and determination of similarity with already identified organisms.

### 2.4. Phylogenetic Analysis

Thirty-one fungi and thirty-two fungal strains were subjected to phylogenetic analysis based on the ITS and *beta-tubulin* sequences, respectively (Supplementary Table S2). The multiple sequences were aligned by ClustalX 2.1 software and the conserved sequences were manually trimmed with GeneDoc for phylogenetic analysis. The phylogenetic analysis was performed by MEGA7 software based on the neighbor-joining (NJ) criteria [18]. Bootstrap analyses were performed to evaluate the robustness of the phylogenies using 1000 replicates for NJ analyses.

### 2.5. Thermotolerance Assay

For the thermotolerance assay, the entomopathogenic fungal isolates (EPF) were cultured on ¼ SDA medium at 25 ± 1 °C in darkness for 10 days. Conidial suspensions were adjusted at 1 × 10^7^ conidia/mL by hemocytometer. The conidial suspensions were exposed at 45 °C for 0, 30, 60, 90, and 120 min. After treatment, 5 μL of spore suspension was dropped on ¼ SDA medium and incubated at 25 ± 1 °C for 18 h. The percentage of the conidia germination was determined by randomly counting the number of germinated and ungerminated conidia in three fields microscopically at 200X under light microscopy [19]. Each treatment was replicated thrice. Conidia were considered as germinated when the length of germ tubes was at least twice the diameter of conidium.

### 2.6. Conidia Productivity

For the conidia productivity, the conidial suspensions were adjusted at 1 × 10^7^ conidia/mL. Further, 10 μL of the sample was dropped on ¼ SDA medium and incubated at 25 ± 1 °C for 7, 10, and 14 days. At each time point, 5 mm of agar block was detached from the center of the colony and put in 1 mL 0.03% Silwet^TM^ in a 1.5 mL tube. The sample was vortexed at 3000 rpm at room temperature for 15 min and the number of conidia was calculated by a hemocytometer under light microscopy [14,15]. Each treatment was replicated thrice.

### 2.7. Ranking of EPF Isolates by Effective Conidia Number (ECN)

The ECN of EPF isolates was determined based on the data of conidia productivity and thermotolerance assay.

### 2.8. Virulence Screening against F. taiwana Last Instar Larvae

Based on the pathogenicity screening against *T. molitor* larvae and ECN ranking, four EPF isolates belonging to the genera *Purpureocillium* and *Fusarium* were selected for primary pathogenicity screening against the last instar larvae of *F. taiwana*. The EPF isolates were cultured on 60 mm of ¼ SDA plates at 25 ℃ for 10 days and the conidia suspensions were prepared as above-mentioned and adjusted to four different concentrations (10^5^ to 10^8^ conidia/mL). Ten larvae of *F. taiwana* were put on a 1% water agar plate for fungal inoculation. The 250 μL conidia suspensions were mixed with 250 μL algae liquid (*Chlorella vulgaris*) [3] and dropped on *F. taiwana* last instar larvae in 1% water agar Petri plate. The mortality of *F. taiwana* was observed and recorded daily until 7 days and the pupation rate and emergence rate were recorded at the same time. Each treatment was replicated three times.

### 2.9. Bioassay of EPF against Different Life Stages of F. taiwana

Based on the pathogenicity screening data, two fungal isolates Pl-NCHU-NPUST-175 and Fv-NCHU-NPUST-178, which showed higher virulence against *F. taiwana* last instar larvae, were selected for bioassay against different life stages of *F. taiwana*. The fungal strains were cultured on ¼ SDA medium at 25 °C for 10 days in a 60 mm plate and the conidia suspensions were prepared as above-mentioned and adjusted to three different concentrations (10^6^,10^7^, and 10^8^ conidia/mL) for the following bioassays. For the bioassay of the egg stage, the 125 μL conidia suspensions were mixed with 250 μL Silwet^TM^ and dropped onto ten eggs of *F. taiwana* on the filter paper on 1% water agar plates and the hatching rate was observed and recorded daily until 10 days. To infect the first to third instar larvae of *F. taiwana*, the 250 μL conidia suspensions were mixed with 250 μL algae liquid and dropped into the 1% water agar plates which had ten larvae of *F. taiwana* on the filter paper and the mortality of *F. taiwana* was observed and recorded daily until 10 days. Each treatment was replicated three times.

### 2.10. Statistical Analysis

The data on the percentage of insect mortality in the bioassay rates were analyzed using Kaplan–Meier Method. Whenever treatments were significantly different (*p* < 0.05), the means were separated using the Tukey test. All analyses were conducted using SPSS ver. 12.1 (SPSS Inc., Chicago, IL, USA, 2009) at a 0.05 (α) level of significance.

## 3. Results

### 3.1. Isolation and Selection of Entomopathogenic Fungi

Six fungal isolates were isolated and cultured in a 1/4 SDA plate. The fungal collection is hereafter given a code from National Chung Hsing University and National Pingtung University of Science & Technology (NCHU-NPUST) fungal collection number. For the primary pathogenicity screening of six fungal isolates against the *T. molitor* larvae, all of the six fungal isolates showed a 100% mortality at 10 days post inoculation (d.p.i.) and, thus, the entompathogenesis was confirmed (Supplementary Figure S1 and Supplementary Table S3). Among these EPFs isolates, NCHU-NPUST-178 showed fast insect-killing activity (100% mortality at 2 d.p.i.), followed by NCHU-NPUST-175 (100% mortality at 3 d.p.i.), 173 (100% mortality at 5 d.p.i.), 176 (100% mortality at 5 d.p.i.), 174 (100% mortality at 6 d.p.i.), and 177 (100% mortality at 8 d.p.i.).

### 3.2. Molecular Identification

**Figure 1:**
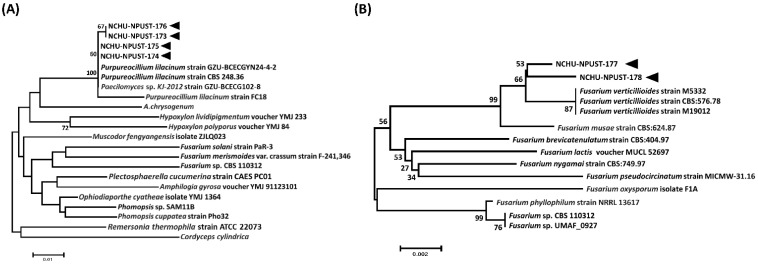
The phylograms of (**A**) *Purpureocillium* spp. (PL-NCHU-NPUST-173 to -176) and (**B**) *Fusarium* spp. (FV-NCHU-NPUST-177 and -178) based on the sequences of the part of *beta-tubulin* gene using the neighbor-joining (NJ) method. Bootstrap support values ≥ 50% are presented above the nodes. The black arrows indicate the fungal isolates used in this study.

All obtained isolates were subjected to molecular identification and phylogenetic analysis. Based on the sequences of the ITS region, six EPF isolates were assigned to two genera, *Purpureocillium* (four isolates, Pl-NCHU-NPUST-173, -174, -175 and -176) and *Fusarium* (two isolates, Fv-NCHU-NPUST-177 and -178) (Supplementary Table S3). Moreover, the identification of species level was also performed based on the sequences of part of the beta-tubulin gene. The results showed that four *Purpureocillium* isolates were identified as *P. lilacinum* and the two *Fusarium* isolates were identified as *F. verticillioides* (Figure 1).

### 3.3. ECN Ranking of the Entomopathogenic Fungi

The results showed that Pl-NCHU-NPUST-176 produced the highest number of conidia (1.39 × 10^8^ conidia/per 5 mm block) followed by Pl-NCHU-NPUST-173 (6.66 × 10^7^ conidia/per 5 mm block), Pl-NCHU-NPUST-175 (5.05 × 10^7^ conidia/per 5 mm block), Pl-NCHU-NPUST-174 (2.55 × 10^7^ conidia/per 5 mm block), Fv-NCHU-NPUST-178 (1.17 × 10^7^ conidia/per 5 mm block), and Fv-NCHU-NPUST-177 (8.95 × 10^6^ conidia/per 5 mm block) after 14 days (Supplementary Figure S2).

**Figure 2:**
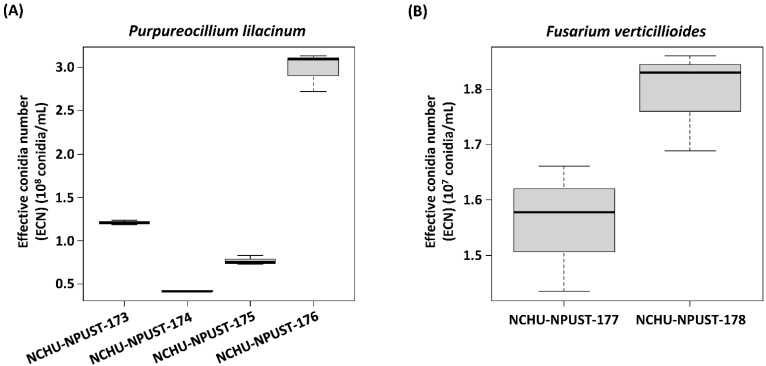
The effective conidia numbers (ECNs) of six fungal isolates. (**A**) *P. lilacinum* PL-NCHU-NPUST-173 to -176; (**B**) *F. verticillioides* FV-NCHU-NPUST-177 and -178.

In the thermotolerance assay, the highest germination rates at 0 min heat treatment were found for Fv-NCHU-NPUST-178 (99.94%), followed by Fv-NCHU-NPUST-177 (99.93%), -175 (99.87%), -174 (99.83%), -173 (99.72%), and -176 (99.14%) (Supplementary Figure S3). After exposure for 30 min, all isolates showed 97.31% to 98.83% conidia germination rate (Supplementary Figure S3). After 60 min of heat treatment, Pl-NCHU-NPUST-173, -174, -175, and -176 had higher conidia germination (96.65% to 98.17%) than Fv-NCHU-NPUST-177 and -178 (89.80% and 87.99%) (Supplementary Figure S3). After exposure for 90 min to 120 min, Pl-NCHU-NPUST-173, -174, -175, and -176 still had high conidia germination (90.56% to 96.57%), while the conidia germination rates of Fv-NCHU-NPUST-177 and -178 were decreased to 67.86% to 74.77% and 58.62% to 73.61% (Supplementary Figure S3). Based on the ECN formula, which was proposed by Chang et al., 2021 [14], the putative survival conidia number under stress was evaluated (Figure 2) and the fungal isolates were ranked by ECN. The ECN rank of *F. verticillioides* isolates is Fv-NCHU-NPUST-178 > 177 and it showed significant differences between Fv-NCHU-NPUST-178 and -177. The ECN rank of *P. lilacinum* isolates was Pl-NCHU-NPUST-176 > -173 > -175 > -174 and they showed significant differences from each other (Figure 2).

### 3.4. Virulence Screening against F. taiwana Larvae

**Figure 3:**
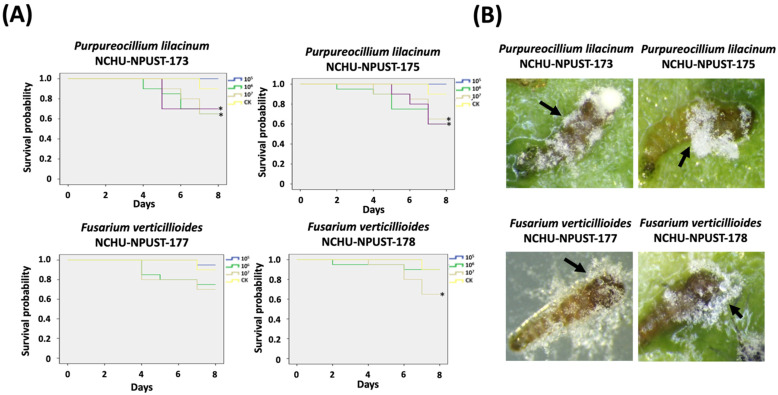
Virulence screening of four selected entomopathogenic fungi isolates against last instar larvae of *F. taiwana*. (**A**) Survival probability of last instar larvae of *F. taiwana* after application of *P. lilacinum* PL-NCHU-NPUST-175 and *F. verticillioides* FV-NCHU-NPUST-178 (* = significant difference based on log-rank test) and (**B**) the mycosis was observed after fungal infection.

Based on the time to cause 100% mortality rate on *T. molitor* larvae and ECN ranking, four EPF isolates (Pl-NCHU-NPUST-173, -175, and Fv-NCHU-NPUST-177, and -178) were selected to bioassay on the last-stage larvae of *F. taiwana*. Pl-NCHU-NPUST-173 and -175 showed significant virulence to the last-stage larvae of *F. taiwana* (5 × 10^7^ to 5 × 10^5^ conidia/mL inoculation, *p* ≤ 0.05) (Figure 3). In addition, Fv-NCHU-NPUST-178 also showed significant virulence to the last-stage larvae of *F. taiwana* (5 × 10^6^ conidia/mL inoculation, *p* ≤ 0.05) (Figure 3). Based on the virulence screening of last-stage larvae, Pl-NCHU-NPUST-175 and Fv-NCHU-NPUST-178 were subjected to bioassay at every stage of *F. taiwana*.

### 3.5. Bioassay of EPF against Different Life Stages of F. taiwana

**Figure 4:**
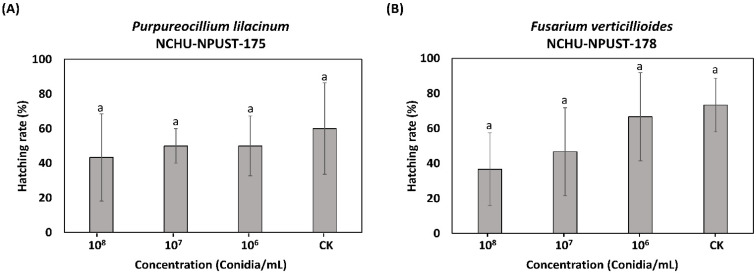
Hatching rate of *F. taiwana* eggs at 7 d.p.i. of (**A**) *P. lilacinum* PL-NCHU-NPUST-175 and (**B**) *F. verticillioides* FV-NCHU-NPUST-178. The same letters mean no significant difference (*p* > 0.05) based on Tukey’s test.

For the egg stage, Pl-NCHU-NPUST-175 revealed the highest ovicidal effects (hatching rate = 43%) at 10^8^ conidia/mL, followed by 10^7^ and 10^6^ conidia/mL (hatching rate = 50%). Fv-NCHU-NPUST-178 showed the highest ovicidal effect (hatching rate = 37%) at 10^8^ conidia/mL, followed by 10^7^ conidia/mL (hatching rate = 47%) and 10^6^ conidia/mL (hatching rate = 67%) (Figure 4).

**Figure 5:**
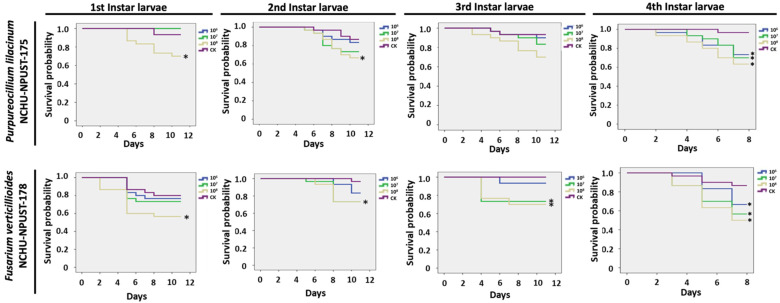
Survival probability of different instar larvae of *F. taiwana* after application of *P. lilacinum* PL-NCHU-NPUST-175 and *F. verticillioides* FV-NCHU-NPUST-178. The survival curves were analyzed based on Kaplan–Meier Method. * = significantly different (*p ≤* 0.05) based on log-rank test.

For the larval stage, two EPFs revealed a dose-dependent effect for both fungal isolates. All the concentrations of Pl-NCHU-NPUST-175 showed significant virulence on last-stage larvae (*p ≤* 0.05) and the highest mortality (38%) in last-stage larvae at 10^8^ conidia/mL and only 10^8^ conidia/mL of Pl-NCHU-NPUST-175 showed significant virulence on first and second instar larvae (*p ≤* 0.05) (Figure 5). Similarly, all the concentrations of Fv-NCHU-NPUST-178 showed significant virulence in last-stage larvae (*p ≤* 0.05) and the highest mortality (50%) in last-stage larvae was caused by a concentration of 10^8^ conidia/mL (Figure 5). In addition, the highest concentration of Fv-NCHU-NPUST-178 also caused significant mortality to all larval stages of *F. taiwana* (Figure 5).

**Figure 6:**
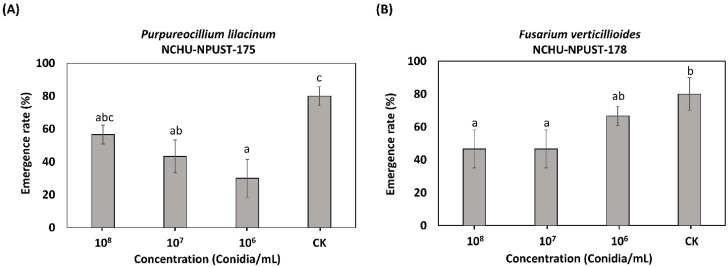
The emergence rate of *F. taiwana* after infection of (**A**) *P. lilacinum* PL-NCHU-NPUST-175 and (**B**) *F. verticillioides* FV-NCHU-NPUST-178 for 7 days. The different letters are significantly different at *p* ≤ 0.05 using Tukey’s test.

For the impact on adult emergency, both EPFs revealed a negative effect (Figure 6). The negative impact on the adult emergency showed a dose-dependent effect in the Fv-NCHU-NPUST-178 treatment and significantly low emergence rates were observed at 10^8^ conidia/mL (46% emergence) and 10^7^ conidia/mL (46% emergence) (Figure 6). For the Pl-NCHU-NPUST-175, a significantly low emergence rate (30%) was observed for an inoculation of 10^6^ conidia/mL, while the emergence rate increased at higher concentrations.

## 4. Discussion

In this study, six fungal isolates were isolated from diseased *F. taiwana* and subjected to the selection system based on Liu et al., 2021. The pathogenicity screening by using mealworm (*T. molitor*) was applied to evaluate the insect-killing activity of these fungal isolates [14,15]. The mortality of the six fungal isolates against mealworms was 100%, indicating the insecticidal potential of the isolates.

Based on sequence data obtained from ITS-rDNA and part of the beta-tubulin gene, these isolates were identified as *Purpureocillium* *lilacinum* (Pl-NCHU-NPUST-173, -174, -175, -176) and *Fusarium* *verticillioides* (Fv-NCHU-NPUST-177 and -178). The ability of these fungal species to infect insects has been previously approved [10,20]. *Purpureocillium* *lilacinum* has been found to infect some species of insects, including Mediterranean fruit fly (*Ceratitis capitata*), nettle caterpillar (*Setora nitens*), cotton aphid (*A. gossypii*), winchuka (*Triatoma infestans*), whitefly (*Bemisia tabaci*), etc. [10,21]. The genus *Fusarium* comprises a large group of species, which are usually found in association with plants and are determined as common plant pathogenic fungi [20]. *Fusarium verticillioides* is a plant pathogenic fungus and infects corn kernels and other tissues [20,22,23]. However, there are reports indicating that this species infects grasshoppers (*Tropidacris collaris*) and *Bemisia tabaci* [20,24,25]. In this study, the ability of *F. verticillioides* isolates to infect the life stages of *F. taiwana* was also demonstrated, indicating its potential for biological control of these pests. However, it is reported that strains of *F. verticillioides* can produce the fumonisin toxin; thus, the safety of Fv-NCHU-NPUST-178 needs to be further considered.

Through the ECN analysis, the potential fungal isolates, which harbor more tolerance to environmental stresses, would be selected for application in the field [14,15]. It is notable that all the tested fungal isolates showed higher thermotolerance compared to the results of the previous studies obtained with *B. bassiana* and *M. anisopliae* [14,26]. Among these fungal isolates, *P. lilacinum* (Pl-NCHU-NPUST-173, -174, -175, -176) showed better results for conidia productivity and thermotolerance than *F. verticillioides* (Fv-NCHU-NPUST-177 and -178), indicating that *P. lilacinum* might have more survival fitness than that of *F. taiwana* in the natural environment. Pl-NCHU-NPUST-176 showed the highest conidia productivity. The conidia productivity may be affected by the genes involved in conidiogenesis, such as the regulator of G-protein signaling (*cag8*) [27]. In addition, there are several different genes that affect conidia production, as shown by *B. bassiana* lacking *Mcm1,* resulting in abnormal germination and reduced conidia production [28].

As aforementioned, the six fungal isolates caused 100% mortality on mealworms and the four selected fungal isolates (Pl-NCHU-NPUST-173, -175, and Fv-NCHU-NPUST-177 and -178) also showed higher larvicidal effect on the last instar larvae of *F. taiwana*, indicating their potential entomopathogenic activity. However, the mortality caused by four fungal isolates was not as high as those on mealworms. The process of EPF infection consists of several stages, including attachment, production of appressorium, production of conidia, germination of conidia, and penetration and invasive growth of the fungal hyphae [28]. It has been reported that the same EPF species have different pathogenicity against different hosts and this might be due to different surface cuticles of hosts, the pH in the gut, etc.; therefore, the difference in pathogenicity of the studied EPF isolates against mealworms and *F. taiwana* larvae is expectable [19].

Based on the bioassay of different life stages of *F. taiwana* by Pl-NCHU-NPUST-175 and Fv-NCHU-NPUST-178, the ovicidal effect was found in both EPFs. Previous studies pointed out that EPFs have the ability to infect insect eggs. For example, the eggs of *Spodoptera frugiperda* and *Spodoptera exigua* were highly susceptible to *M. anisopliae* and *B. bassiana* infection [29]. For the larval stage, two EPFs revealed a dose-dependent effect for both fungal isolates. The results showed the lethal effect to the last instar larvae of *F. taiwana*, but their efficacy is low to other life stages of *F. taiwana*. It has been reported that different fungal isolates showed different virulence against different stages of insects. For example, after infection of *Bactrocera zonata* and *Bactrocera dorsalis* with EPFs, adults were found to be more susceptible than larvae and pupae were more resistant than larvae [30]. This might be due to molting, which led to the loss of inoculum and, hence, lower chances of fungal infection [31]. Moreover, it also found that different fungal isolates showed varying inherent potential to infect different life stages of target insects. For example, the third and fourth *Culex quinquefasciatus* larvae were highly susceptible to *Fusarium oxysporum*, but the *B. bassiana* and *M. anisopliae* are highly effective in reducing larval survival and adult emergence [32]. It was noted that the emergence rate of *F. taiwana* adults was influenced by the EPF inoculation. There are also related reports showing that fungi have an impact on the adult emergence rate. The emergence rate of *Galleria* *mellonella* infected with different concentrations of *P. lilacinus* and *B. bassiana* was increasingly decreased with higher fungal concentrations [33]. In conclusion, according to the results of this study, Pl-NCHU-NPUST-175 and Fv-NCHU-NPUST-178 showed the potential to be integrated into the management strategies of *F. taiwana* populations.

## 5. Conclusions

*F. taiwana* is a serious pest in Taiwan, so it is important to find effective and environmentally friendly ways to control it. In this study, two different fungal isolates, Pl-NCHU-NPUST-175 and Fv-NCHU-NPUST-178, were compared. It was found that Fv-NCHU-NPUST-178 had a better effect on the eggs and larvae of different instars of *F. taiwana* in pathogenicity tests, but in the ranking of ECN, Pl-NCHU-NPUST-175 was better than Fv-NCHU-NPUST-178. It has also been reported that *F. verticillioides* can produce fumonisin toxin [22]; therefore, the risk assessment needs to be further considered when it is applied to the environment. Therefore, compared with Fv-NCHU-NPUST-178, Pl-NCHU-NPUST-175 is more suitable for direct and large-scale spraying in the field to achieve the purpose of prevention and control. On the other hand, despite Fv-NCHU-NPUST-178, which showed higher environmental tolerance, the issue of the fumonisin toxin needs to be carefully addressed. In this study, Pl-NCHU-NPUST-175 and Fv-NCHU-NPUST-178 showed potential ability to control *F. taiwana* and provided alternative materials for the integrated past management of *F. taiwana*.

## Data Availability

All sequences data were submitted to NCBI.

## Supplementary Materials

The following supporting information can be downloaded at: https://www.mdpi.com/article/10.3390/jof8080861/s1. Table S1: Primer sets for fungal molecular identification; Table S2: The ITS and beta-tubulin sequences of entomopathogenic fungi from NCBI Genbank. Table S3: Pathogenicity screening of six selected fungal isolates against meal worms. Figure S1: Pathogenicity screening of 6 fungal isolates. (A) Survival rate of mealworms. Each fungal isolate was repeated for six times; (B) the symptom of fungal mycosis was observed; Figure S2: Conidial productivity of 6 fungal isolates. (A) Four fungal isolates of *Purpureocillium lilacinum* and (B) Two fungal isolate of *Fusarium verticillioides*. The data were collected after 7, 10 and 14 days cultivation. Total three blocks (5 mm in diameter) were calculated for each fungal isolate. The experiments were replicated three times for each fungal isolate; Figure S3: Thermotolerance of 6 fungal isolates under different durations of 45 °C heat treatment. (A) Four fungal isolate of *Purpureocillium lilacinum* and (B) Two fungal isolate of *Fusarium verticillioides*. The data were collected after 0, 30, 60, 90 and 120 min heat treatment. The experiments were replicated three times for each fungal isolate.
