# Supplementary figures and images for: Evaluation of the Potential Entomopathogenic Fungi Purpureocillium lilacinum and Fusarium verticillioides for Biological Control of Forcipomyia taiwana (Shiraki)
Source: J Fungi (Basel). 2022 Aug 16;8(8):861. doi: 10.3390/jof8080861 (PMC9410248; doi:10.3390/jof8080861)

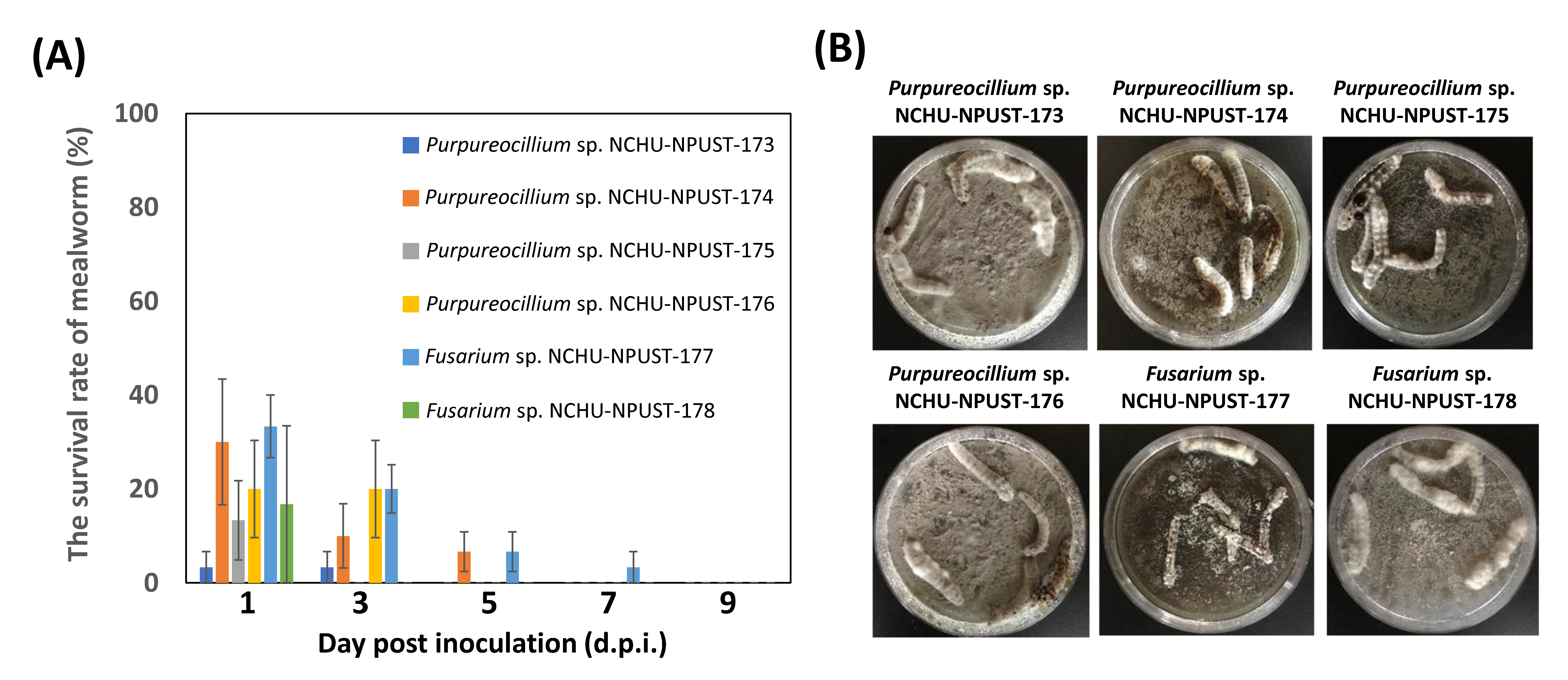

Supplement: Supplementary file 1 [file jof-08-00861-s001.zip › Supplementary Figure S1.tiff]

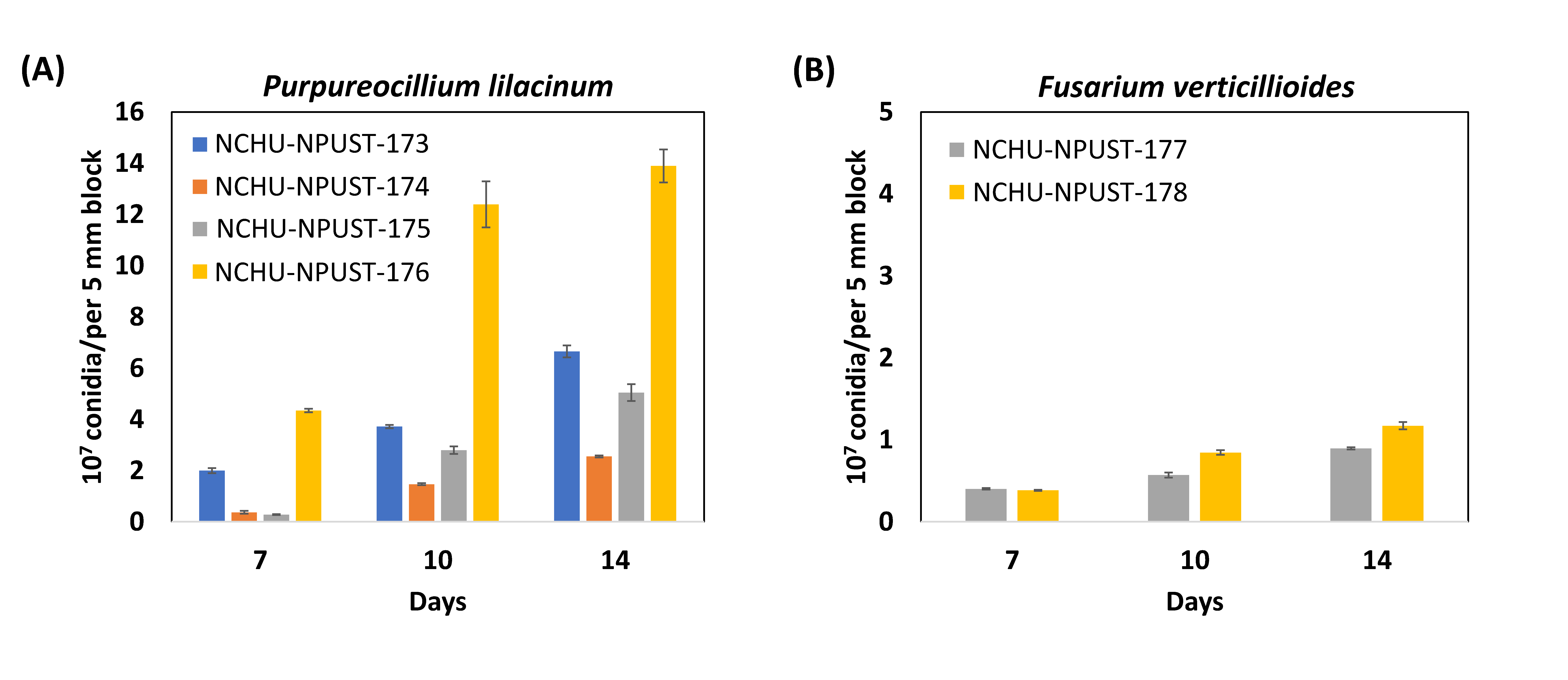

Supplement: Supplementary file 1 [file jof-08-00861-s001.zip › Supplementary Figure S2.tiff]

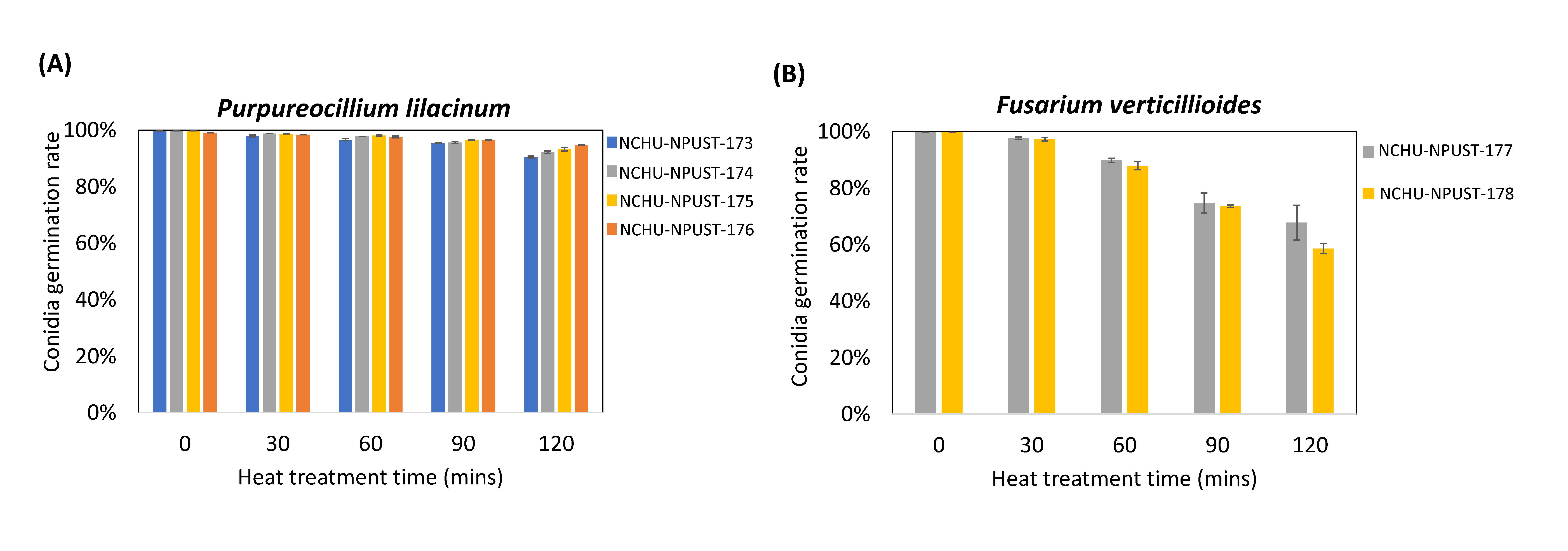

Supplement: Supplementary file 1 [file jof-08-00861-s001.zip › Supplementary Figure S3.tiff]
